# Efficacy of Caffeic Acid on Diabetes and Its Complications in the Mouse

**DOI:** 10.3390/molecules26113262

**Published:** 2021-05-28

**Authors:** Nada Oršolić, Damir Sirovina, Dyana Odeh, Goran Gajski, Vedran Balta, Lidija Šver, Maja Jazvinšćak Jembrek

**Affiliations:** 1 Department of Animal Physiology, Faculty of Science, University of Zagreb, Rooseveltov trg 6, HR-10000 Zagreb, Croatia; damir.sirovina@biol.pmf.hr (D.S.); dyana.odeh@biol.pmf.hr (D.O.); vedran.balta25@gmail.com (V.B.); 2 Mutagenesis Unit, Institute for Medical Research and Occupational Health, HR-10000 Zagreb, Croatia; ggajski@imi.hr; 3 Department of Biochemical Engineering, Faculty of Food Technology and Biotechnology, University of Zagreb, Pierottijeva 6, HR-10000 Zagreb, Croatia; lsver@pbf.hr; 4 Laboratory for Protein Dynamics, Division of Molecular Medicine, Ruđer Bošković Institute, Bijenička Cesta 54, HR-10 000 Zagreb, Croatia; maja.jazvinscak.jembrek@irb.hr; 5 Department of Psychology, Catholic University of Croatia, Ilica 242, HR-10000 Zagreb, Croatia

**Keywords:** alloxan, diabetes, mice, caffeic acid, blood, liver and kidney damage, atherogenic indices

## Abstract

Diabetic dyslipidemia and hyperglycemia contribute to excessive reactive oxygen species (ROS) production, leading to deleterious complications, such as nephropathy, atherosclerosis and cardiac dysfunction, and target major organs in the body. The aim of this study was to investigate the effect of caffeic acid (CA) on mouse weight and survival, serum level of fasting blood glucose (FBG), serum lipid parameters and atherogenic indices, oxidative damage in blood, liver and kidney tissue, pathophysiological changes and their function markers in healthy and alloxan-induced type 1 diabetic mice. Diabetes was induced in mice with a single intravenous injection of alloxan (75 mg kg^−1^). Two days later, CA (50 mg kg^−1^) was given intraperitoneally for seven days in diabetic mice. Diabetes affected glucose level, lipid profile, hematological and biochemical parameters, induced DNA damage and apoptotic/necrotic death in whole blood cells, liver and kidney, leading to weight loss and a decreased lifespan. CA treatment of diabetic mice revealed a protective effect on the liver and kidney, hypoglycemic and hypolipidemic properties and high protection against atherogenic outcomes. The obtained results suggest that CA is a safe and potent agent against diabetes that acts as an effective antioxidant in reducing serum glucose, lipid profile and atherogenic indices, leading to increased lifespan in mice.

## 1. Introduction

Diabetes mellitus (DM) is characterized by high blood sugar (hyperglycemia), dysregulated carbohydrate and lipid metabolism and oxidative stress, coupled with a high incidence of microvascular disorders [1,2]. The number of people suffering from diabetes is steadily increasing, with a global prevalence in 2017 of 451 million people and an expected increase to 693 million by 2045 [3,4]. Detailed metabolic analyses revealed 12–61% and up to 20% lower whole-body (skeletal muscle) and hepatic insulin sensitivity, respectively, in type 1 diabetes (T1D), depending on the population studied. T1D patients exhibit impaired muscle adenosine triphosphate (ATP) synthesis and enhanced oxidative stress, predominantly related to hyperglycemia [5]. 

T1D is a chronic autoimmune metabolic disease characterized by an absolute insulin deficiency caused by T-cell–mediated autoimmune destruction of pancreatic β-cells, leading to disturbed glucose metabolism. T1D accounts for 7–12% of the total diabetes population and leads to long-term complications including cardiovascular disease (CVD which are at least 10-fold higher in T1D compared with non-diabetic age population [6]. Death caused by CVD is roughly 2-4 times more frequent among type 1 diabetes mellitus (T1DM) population compared with their non-diabetic counterparts [7]. In addition, diabetic nephropathy (DN), a microvascular complication in T1D, leads to a 20–50% probability of developing end-stage renal disease, while selective insulin resistance and lipotoxicity, reported to be important aspects of end-organ damage in T1D, could explain the observed chronic inflammation in atherosclerosis and diabetic kidney disease [8]. According to epidemiological data, impaired glycemic control, lipoprotein disturbances, oxidative stress and inflammation are important parameters that can accelerate atherosclerosis. Thus, in a large cohort study, an increase of 1 mmol L^−1^ of LDL-cholesterol (LDL-c) was associated with a 35% to 50% greater risk of overall CVD [9]. Except for LDL-c, elevated serum triacylglycerol (TG), total cholesterol (TC) and very-low-density lipoprotein-cholesterol (VLDL-c) concentrations out of their reference intervals are often indications of dyslipidemia and an increased incidence of CVD. Furthermore, the same data revealed that the risk of atherogenic dyslipidemia was higher by two- to three-fold in DM patients [10]. Atherogenic dyslipidemia in DM, especially elevated serum LDL-c and TG concentrations, are major factors implicated in eliciting a higher frequency of cardiovascular events [10]. More precisely, atherogenesis is a process in vascular tissues, facilitated by cellular and acellular elements and resulting in the formation of atheromatous plaques on the inner walls of arteries, which causes the narrowing of blood flow channels as well as the hardening of artery walls (atherosclerosis) with lesions of the coronary and blood vessels responsible for the pathogenesis of various cardiovascular and cerebrovascular diseases. 

Alloxan, the most popular diabetogenic agent in experimental animals, induces T1D by several processes: the oxidation of –SH groups, inhibition of glucokinase, generation of free radicals and disturbances in calcium homeostasis [11,12,13]. In addition, alloxan, as a 5,5-dihydroxyl pyrimidine-2,4,6-trione, is a carcinogen and glucose analog toxic to β-cells. The pathological effect of alloxan is based on the selective inhibition of glucose-stimulated insulin secretion and ROS formation through the alloxan redox cycle, leading to selective apoptosis and necrosis in pancreatic β-cells. Furthermore, impaired red blood cell (RBC) deformability due to oxidative stress plays a key role in the pathogenesis of the chronic vascular complications of DM and renal failure progression [2,11,12,13,14,15,16]. Oxidative DNA damage associated with ROS include apurinic/apyrimidinic (abasic) DNA sites, oxidized purines and pyrimidines, and single-strand breaks (SSBs) and double-strand breaks (DSBs) in different tissues, including lymphocytes, kidney, heart, eyes, nerves, liver, small and large vessels, and the gastrointestinal system [2,11,14,15,16,17,18]. ROS may also damage DNA indirectly, through a reaction with lipids, proteins and other cellular components that produces electrophilic species that can react with DNA, leading to the development of diseases such as aging, cancer, CVD, immune system diseases and degenerative diseases [14,15,16,17,18]. 

The effective control of hyperglycemia via natural antioxidants may be important in reducing diabetes complications, especially micro- and macro-vascular diseases. Plants used in traditional medicine to treat diabetes represent a valuable alternative for the management of this disease, due to their low cost, high efficiency and few side effects. Thus CA, present in propolis from honeybee hives [2], many vegetables, fruits and beverages, such as carrots, tomatoes, strawberries, blueberries, olive oil, coffee, artichokes, potatoes, chicory, pears, apples, kiwis, cherries and plums, among others [19], has been found to possess a wide range of pharmacological activities. Dietary polyphenols, including CA, may inhibit α-amylase and β-glucosidase, glucose absorption in the intestine by sodium-dependent glucose transporter 1 (SGLT1), stimulate insulin secretion and reduce hepatic glucose output. Polyphenols may also enhance insulin-dependent glucose uptake, activate adenosine monophosphate-activated protein kinase (AMPK), modify the microbiome and have anti-inflammatory, antimicrobial, immunomodulatory, anti-aging, antioxidant, antidiabetic and hypocholesteremic activities [2,12,20,21].

Natural phenols such as CA may achieve their protective role through several mechanisms, such as activating and protecting intercellular antioxidant enzymes as well as through hydrogen atom transfer, single electron transfer and metal chelation [22,23]. Another protective mechanism of polyphenols is their capacity to inhibit the activation of nuclear transcription factor-κB (NF-κB) signaling pathways and upregulate the transcription factor NrF2 (nuclear erythroid 2-related factor 2), which binds to the antioxidant response element (ARE) and thereby regulates the expression of more than 200 genes involved in the cellular antioxidant and anti-inflammatory defense, which may be important in the detoxification of xenobiotics such as alloxan [24]. The antioxidant, anti-inflammatory, anticoagulatory and antihyperglycemic effect of CA was confirmed in C57BL/KsJ-db/db mice and in streptozotocin-induced diabetes in Balb/c mice and rats [25,26,27].

Given that the risk of atherogenic dyslipidemia is increased two to three times in patients with diabetes and coronary and vascular lesions, we hypothesize that, by improving the understanding of lipoprotein disorders, reducing hyperglycemia, and controlling glucose levels, as well as insulin deficiency or resistance, we can help to combat CVD in T1D.

In the presented work, we examined the antioxidative effect of CA in vivo and its possible protective effect on mouse weight and survival, the serum level of fasting blood glucose (FBG), serum lipid parameters and atherogenic indices, as well as liver and kidney functions in healthy and alloxan-induced type 1 diabetic mice. In addition, we evaluated the oxidative damage induced by alloxan in mouse blood, liver and kidney tissue and the protective effect of CA on DNA damage by alkaline comet assay, a quick and sensitive method for the detection of DNA damage and incomplete excision repair sites, as well as antioxidant cell status and pathophysiological changes in the liver and kidney and markers of their functions. 

## 2. Results

### 2.1. Effect of Caffeic Acid on the Body Weight of Alloxan-Induced Diabetic Mice

Weight loss, which is one of the clinical features of DM, may be due to the degeneration of adipocytes and muscle tissues to make up for the energy lost from the body due to frequent urination and the overconversion of glycogen to glucose. The result of the effect of CA on the body weight of mice with alloxan–induced diabetes is shown in Table 1. 

Alloxan at a dose of 75 mg kg^−1^ *iv* successfully causes diabetes in mice; blood glucose level was elevated on the second day after treatment in each mouse, ranging between 11.5 and 16.75 mmol L^−1^ (13.41 ± 1.51). On the 10th day of all treatments, the body weight of diabetic mice demonstrated significant weight loss when compared with the control mice; the decrease was the largest between the 3rd and 10th day (−3.75 to −12.10%), and at the end of the 45th day, there was a significant difference in the weight of the control and the diabetic untreated mice. The weight gain in the control animals was 12.75%, whereas in diabetic animals, a decrease of 13.63% was observed. The weight of healthy CA-treated animals was slightly lower compared to the healthy control mice. In diabetic animals treated with CA, body weight started to recover easily and reached a mass similar to that of healthy (nondiabetic control) animals. During the experiments, four animals from the diabetic control group died, while diabetic animals treated with CA all survived (data not shown). 

### 2.2. Effect of Alloxan on Blood Glucose Level and the Hypoglycemic and Antihyperglycemic Activity of a Single Dose of Caffeic Acid in Alloxan-Induced Diabetic Mice

The effect of a single dose of caffeic acid on normoglycemic healthy and hyperglycemic alloxan-induced diabetic mice is summarized in Table 2. 

There was no significant difference in baseline FBG (at 0 h) between two normoglycemic mice, but the hypoglycemic effect became significant (*P* < 0.05) following CA administration, reaching its maximal peak four hours later. Visible changes in glucose levels were observed in CA-treated mice after 4 h (7.23) in relation to FBG before treatment (0 h). 

**Figure 1:**
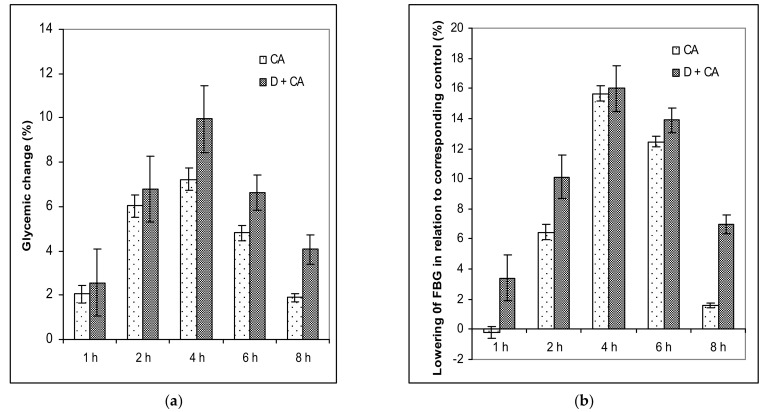
(**a**) The effect of a single dose of caffeic acid on glycemic variation (%) within a group of healthy and alloxan-induced diabetic mice. The percentage of glycemic variation within a group was calculated as a function of time (t) by applying the formula: % glycemic change = (G_0_ − G_1_) × 100/G_0_, where G_0_ and G_1_ represent glycemic values before and at one, two, four, six and eight hours after caffeic acid treatment, respectively. (**b**) The decrease of blood glucose levels in relation to the corresponding control was calculated according to the formula described below. The decreased percentage of blood glucose level = (1 − Ge/Gc) × 100, where Ge and Gc represent the blood glucose concentration in CA-treated healthy or CA-treated diabetic mice and corresponding control mice (healthy control or alloxan-induced diabetic mice), respectively. Diabetic mice were injected *iv* with alloxan at a single dose of 75 mg kg^−1^ body weight. The treatment of healthy control animals or diabetic animals with a single dose of caffeic acid started on the second day after the induction of diabetes with a dose of 50 mg kg^−1^ *ip*; *n* = 9 for each treatment. Abbreviations: CA, caffeic acid-treated healthy animals; D + CA, diabetic group treated with C.

Despite the lack of an initial difference in alloxan-induced hyperglycemic mice, the treatment of diabetic mice with CA significantly decreased glucose levels after 4 h (*P* < 0.05) compared to the diabetic model. FBG levels were reduced by 9.95% in relation to FBG before treatment (0 h) (Table 2). The percentage of glycemic variation after treating normoglycemic and hyperglycemic mice with CA is presented in Figure 1a. 

Compared with the appropriate control, CA reduced glucose levels in healthy control groups after 2, 4 and 6 h by 4.71%, 5.27% and 3.15%, respectively. At the same time, in the in CA-treated diabetic mice, the reduction of FBG levels compared with the diabetic model, was after 2 h (6.28%), 4 h (11.67%), 6 h (7.88%) and 8 h (4.93%) respectively (Figure 1b).

### 2.3. Antihyperglycemic Activity of a Repeated Dose of Caffeic Acid in Alloxan-Induced Diabetic Mice

**Figure 2:**
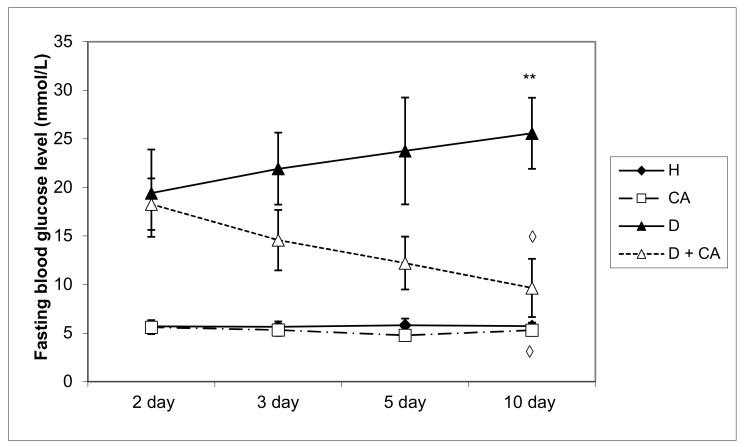
The antihyperglycemic effect of a repeated dose of caffeic acid on blood glucose levels in healthy and alloxan-induced diabetic mice. Diabetic mice were injected *iv* with alloxan at a single dose of 75 mg kg^−1^ body weight. The treatment of diabetic animals with caffeic acid started on the second day after the induction of diabetes with a dose of 50 mg kg^−1^ *ip* for 7 consecutive days. Results are presented as mean fasting blood glucose (FBG) level; *n* = 9 for each treatment. ∗ Statistically significant compared to healthy (H) control group (∗∗ *P* < 0.01). ^◊^ Statistically significant compared to diabetic (D) control group (^◊^ *P* < 0.05; ^◊◊^ *P* < 0.01). Abbreviations: H, control group (normal healthy animals); CA, caffeic acid-treated healthy animals; D, diabetic control group (diabetic model animals); D + CA, diabetic group treated with CA.

The FBG level in alloxan-induced diabetic mice was 7.71 mmol L^−1^ higher (13.41 versus 5.7 mmol L^−1^) than that of the normal control animal group (0 h, the time before treatment). On the 10th day, the FBG level of diabetic mice was increased by 13.68 mmol L^−1^ compared to the FBG of normal control mice. The treatment of diabetic mice with CA resulted in a 50.28% reduction in FBG concentration in relation to diabetic mice on the 10th day. The glucose levels in the CA-treated mice on day 10 were not significantly changed compared to the control group (Figure 2).

### 2.4. Effect of Caffeic Acid on the Hematological and Biochemical Parameters of Liver and Kidney Function

The RBC count, hematocrit (Hct) and hemoglobin (Hb) of untreated diabetic mice showed a significant decrease (*P* < 0.05) compared to normal control mice, while the other parameters did not change, except for an increase in MCV (Table 3). CA supplementation to diabetic mice significantly reversed these parameters. 

As markers of hepatic damage, the ALP, AST and ALT levels were increased significantly in diabetic mice (*P* < 0.05) compared to the healthy control group. LDH and urea levels were most altered (*P* < 0.01) in alloxan-treated animals in relation to controls. The total protein value in alloxan-induced diabetic mice was decreased compared with normal control mice (Table 4). The treatment of diabetic mice with CA returned these values to almost similar or slightly higher levels than in the control group.

### 2.5. Effect of Caffeic Acid on Erythrocyte Osmotic Fragility and Erythrocyte Hemolysis

**Figure 3:**
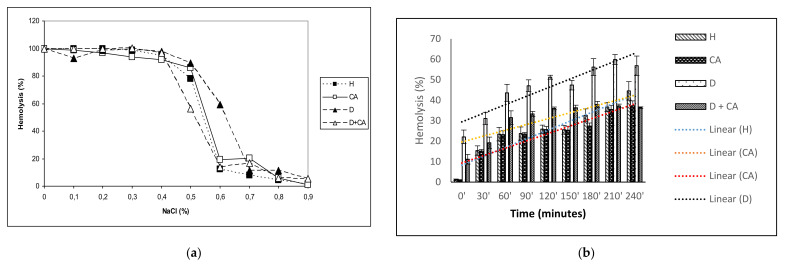
(**a**) Effect of caffeic acid on the osmotic fragility of erythrocytes in healthy and alloxan-induced diabetic mice. Diabetic mice were injected *iv* with alloxan at a single dose of 75 mg kg^−1^ body weight. The treatment of diabetic animals with caffeic acid started on the second day after the induction of diabetes with a dose of 50 mg kg^−1^ *ip* for 7 consecutive days. (**b**) Linear representation of hemolysis curve for the effect of caffeic acid in healthy and alloxan-induced diabetic mice. Abbreviations: H, control group (normal healthy animals); CA, caffeic acid-treated healthy animals; D, diabetic control group (diabetic model animals); D + CA, diabetic group treated with CA.

Oxidative-stress-related diseases, such as diabetes, are known to be associated with changes in erythrocyte morphology via the oxidation or glycation of membrane and cytoskeletal proteins. Oxidative stress alters membrane structure and function, leading to an increase in the osmotic fragility of the erythrocyte membrane and a decrease in cellular fluidity. The results in Figure 3a show the osmotic fragility curve of the erythrocytes of a normal and a diabetic group of mice exposed to treatment with CA. The 50% hemolysis of erythrocytes in the control group (H) was at 0.55% of NaCl concentration, while in the diabetic group 50% hemolysis of erythrocytes occurred at 0.63%. In the diabetic group treated with CA, 50% hemolysis of erythrocytes occurred at 0.53% NaCl, while normal mice treated with CA had 50% hemolysis at 0.55% NaCl. 

ROS generation is increased in diabetes due to prolonged exposure to hyperglycemia. Insufficient antioxidant defenses could be reduced by treating animals with CA. Our hemolysis results show that erythrocytes are sensitive to oxidative damage, especially the erythrocytes of mice with diabetes, where the level of hemolysis is significantly higher than that of CA-treated diabetic mice and control mice (Figure 3b). It seems that the released hemoglobin reacts with H_2_O_2_, leading not only to the oxidized form but also to the degradation of the heme, releasing Fe, which, in reaction with H_2_O_2_, increases the level of oxidative stress, leading to increased erythrocyte degradation. After adding a trendline to the obtained erythrocyte sensitivity results, it was observed that 50% of erythrocyte hemolysis occurred in diabetic mice after 150 min of incubation with H_2_O_2_, while treatment of diabetic mice with CA and control mice achieved 30–35% erythrocyte hemolysis after 150 min.

### 2.6. Effect of Caffeic Acid on Lipid Parameters and Atherogenic Indices in Alloxan–Induced Diabetic Mice

Hyperglycemia is the dominant risk factor for CVD in T1D. Diabetic mice showed a significant increase (*P* < 0.01) in the blood levels of TG, total cholesterol, LDL, VLDL-c and parameters of atherogenic indices (Table 5) in relation to healthy control mice. On the other hand, HDL-c was significantly reduced in untreated diabetic mice. As seen in Table 5, there was a significant difference between untreated diabetic mice and CA-treated diabetic mice; CA treatment decreased the blood levels of TC and TG by approximately 22% and LDL and VLDL by 37.56% and 21.70%, respectively, while HDL increased by 63% when compared to the diabetic control mice. 

All atherogenic parameters were reduced: AIP by ~40.5%, AC by ~58.5%, CRR and TG/ HDL-c ratio by 47.5 and 52.5%, respectively, when compared to diabetic mice, while CPI increased by more than 2× (138%) in CA-treated diabetic mice (Table 5). There was no significant difference in lipid parameters between the healthy control group and healthy CA-treated animals (Table 5). The atherogenic protection of CA-treated diabetic mice was 58.5%.

### 2.7. Effect of Caffeic Acid on Lipid Peroxidation

**Figure 4:**
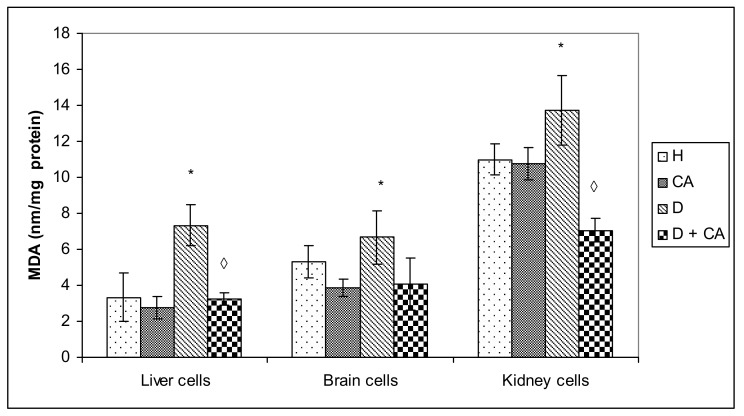
Effect of caffeic acid on lipid peroxidation in liver, brain and kidney in healthy and alloxan-induced diabetic mice. Diabetic mice were injected *iv* with alloxan at a single dose of 75 mg kg^−1^ body weight. The treatment of diabetic animals with caffeic acid started on the second day after the induction of diabetes with a dose of 50 mg kg^−1^ *ip* for 7 consecutive days. Results are presented as mean ±SEM; *n* = 6 for each treatment. ∗ Statistically significant compared to the healthy (H) control group (∗ *P* < 0.05). ^◊^ Statistically significant compared to diabetic (D) control group (^◊^ *P* < 0.05). Abbreviations: H, control group (normal healthy animals); CA, caffeic acid-treated healthy animals; D, diabetic control group (diabetic model animals); D + CA, diabetic group treated with CA; MDA, malondialdehyde.

The principle behind the lipid peroxidation assay is the reaction of one of the end-products of lipid oxidation, malondialdehyde (MDA), with thiobarbituric acid (TBA). The levels of MDA in the liver, brain and kidney of untreated diabetic animals and animals treated with caffeic acid are presented in Figure 4. 

As presented in Figure 4, diabetes caused a significant increase (*P* < 0.05) in MDA in the liver and brain of diabetic animals. The treatment of diabetic animals with CA caused a notable decrease in lipid peroxidation levels in the liver and kidney as observed by a drop in MDA concentrations to values similar to those observed in healthy mice (*P* > 0.05). In addition, cerebral tissue was affected by oxidative changes but to a lesser extent, and the difference between MDA levels in diabetic and healthy mice did not reach statistically significant levels. However, the treatment of diabetic animals with CA still lowered the observed MDA levels in brain tissue compared to untreated animals (Figure 4). The treatment of healthy mice with CA reduced MDA levels in all tissues but without a statistically significant difference compared to healthy animals.

### 2.8. Effect of Caffeic Acid on the Alkaline Comet Assay

**Figure 5:**
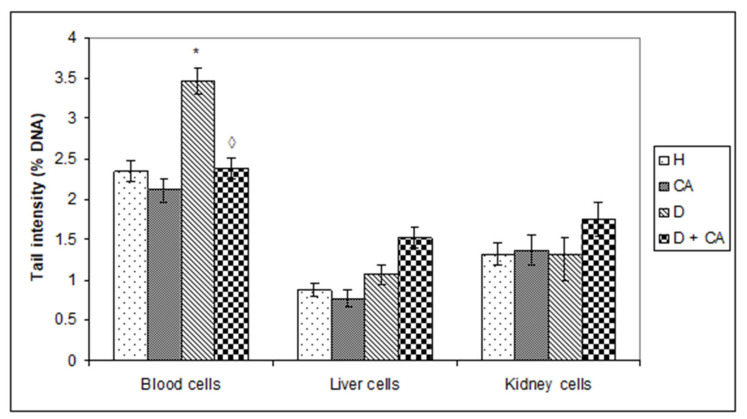
Effect of caffeic acid on DNA damage in peripheral blood cells, liver and kidney cells in healthy and alloxan-induced diabetic mice. Diabetic mice were injected *iv* with alloxan at a single dose of 75 mg kg^−^^1^ body weight. The treatment of diabetic animals with caffeic acid started on the second day after the induction of diabetes with a dose of 50 mg kg^−^^1^ *ip* for 7 consecutive days. Results are presented as mean ± SEM; *n* = 4 for each treatment. ∗ Statistically significant compared to healthy (H) control group (∗ *P* < 0.05). ^◊^ Statistically significant compared to diabetic (D) control group (^◊^ *P* < 0.05). Abbreviations: H, control group (normal healthy animals); CA, caffeic acid-treated healthy animals; D, diabetic control group (diabetic model animals); D + CA, diabetic group treated with CA.

The results of the alkaline comet assay parameters measured in whole blood cells, liver and kidney cells are presented in Figure 5. 

Statistical analyses showed that alloxan treatment, when in whole blood cells are measured, increased DNA damage compared to the control (nondiabetic) animals (*P* < 0.05); the results show that alloxan increased the percentage of DNA in tails (tail intensity) by 47.23% (3.46 ± 0.16 versus 2.35 ± 0.13) in relation to control animals. Decreased DNA damage was seen in CA-treated diabetic mice compared to the alloxan treatment (diabetic mice) by 45.37%, while, in relation to nondiabetic control mice, the percentage of DNA in tails was increased by 1.27%. In the liver and kidney cells, alloxan treatment did not induce an increase in DNA damage between the control group, diabetic and CA-treated diabetic mice, but a significantly increase in the percentage (%) of apoptosis and necrosis in histopathological analysis.

### 2.9. Histopathological Observation

**Figure 6:**
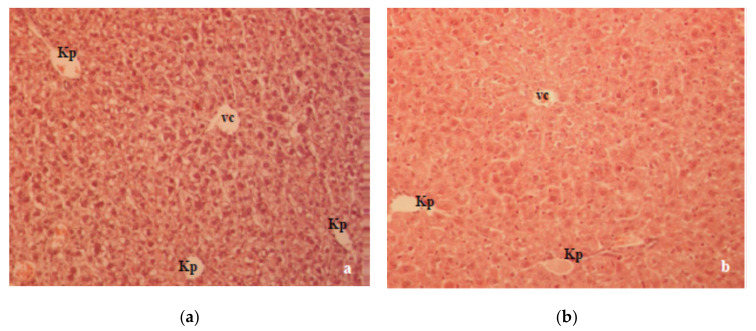
(**a**) In the liver of diabetic mice, almost all cells were considerably vacuolated in the central and peripheral part of the individual lobules. Hepatocytes underwent dystrophic changes (granular dystrophy). In some samples, lympholeucocytal infiltration foci with the necrobiosis, necrosis and apoptosis of hepatocytes were present. (**b**) In mice treated with CA, liver cells around the centralis (vc) vein were more strongly vacuolated than cells around the Kiernan space (Kp), with the degree of cell vacuolization being lower compared to diabetic controls. Abbreviations: vc-vena centralis; Kp-Kiernan space.

**Figure 7:**
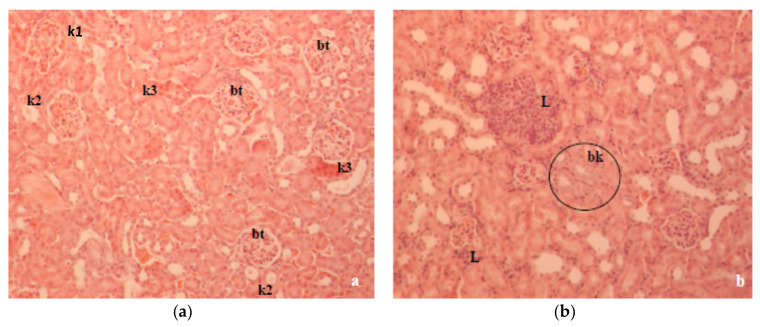
Kidney tissue of diabetic mice stained with hematoxylin and eosin (HE). (**a**) Control diabetic mice (200×). The intercapsular spaces of most renal corpuscles (bt) were narrowed, and the outer leaves of the Bowman’s capsule thickened. The changes were also visible on the channels. Channels with a vacuolated cytoplasm, channels with intraluminal vacuoles (k1), channels with thinned epithelium (k2) with or without intraluminal smaller or larger eosinophilic mass, and eosinophilic channels (k3) are signs of damage to the epithelium. (**b**) Diabetic mice treated with CA. We observed more smaller or larger clusters of lymphocytes (L), more tubules with thinned epithelium, fewer eosinophilic tubules and more basophilic tubules (bk) in the renal cortex compared to diabetic controls.

The histology of the liver and kidney is presented in Figure 6 and Figure 7, respectively. Histopathological observation of the liver sections of alloxan-induced diabetic mice showed several lesions, including cellular vacuolization, cytoplasmic eosinophilia and lymphocyte infiltrations, but with individual variability. The presence of single or small foci of eosinophilic cells and pale foci was another characteristic of damaged cells indicating apoptosis and necrosis (Figure 6a). In CA-treated diabetic mice, the degree of cell vacuolization was lower compared to the diabetic control. CA treatment helped to improve the normal arrangement of the liver and it reduced inflammation (Figure 6b).

**Figure 8:**
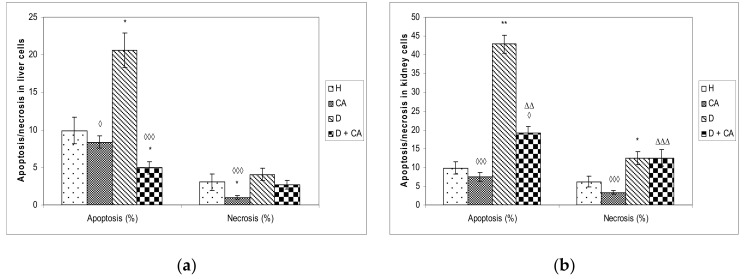
Effect of caffeic acid on apoptosis and necrosis death in the liver (**a**) and kidney (**b**) cells in healthy and alloxan-induced diabetic mice. Diabetic mice were injected *iv* with alloxan at a single dose of 75 mg kg^−1^ body weight. The treatment of diabetic animals with caffeic acid started on the second day after the induction of diabetes with a dose of 50 mg kg^−1^ *ip* for 7 consecutive days. Results are presented as mean ±SEM; *n* = 4 for each treatment. ∗ Statistically significant compared to healthy (H) control group (∗ *P* < 0.05; ** *P* < 0.01). ^◊^ Statistically significant compared to diabetic (D) control group (^◊^ *P* < 0.05; ^◊◊◊^ *P* < 0.001). ^Δ^ Significantly different from the CA group (^ΔΔ^ *P* < 0.01; ^ΔΔΔ^ *P* < 0.001). Abbreviations: H, control group (normal healthy animals); CA, caffeic acid-treated healthy animals; D, diabetic control group (diabetic model animals); D + CA, diabetic group treated with CA.

Apoptotic and necrotic cells were also found in the liver and kidney tissues of all groups. As shown in Figure 8, alloxan, compared to controls, caused a significant increase in the percentage of apoptotic and necrotic cell death in liver cells (apoptosis 20.63 versus 9.91; necrosis 4.04 versus 3.02) and kidney cells (apoptosis 42.86 versus 9.85; necrosis 12.50 versus 6.22). In CA-treated diabetic mice, the percentage of apoptotic and necrotic cell death decreased to 4.96% and 2.70 in liver cells (Figure 8) and in kidney cells to 19.17% cells for apoptosis and 12.50% cells for necrosis. No increase in apoptosis or necrosis was observed in peripheral blood lymphocytes (data not shown).

## 3. Discussion

Diabetes mellitus is a complex metabolic disease characterized by hyperglycemia, glycosuria and several microvascular and macrovascular complications. Alloxan, a diabetic agent, successfully caused significant biochemical, histopathological and functional changes in the blood, liver and kidney, such as the elevation of BGL; TG; TC; LDL; VLDL cholesterol; liver enzymes; serum blood urea; MDA level; and DNA damage in peripheral blood cells, liver and kidney, and a significant decrease in serum proteins, HDL and body weight. The treatment of diabetic mice with CA revealed a protective effect on the liver and kidneys and hypoglycemic and hypolipidemic properties and suggests that CA is a safe and potent antioxidant in diabetes that effectively reduces serum glucose, LDL and atherogenic indices leading to long-term diabetes control and animal survival. High blood sugar levels clearly indicate persistent hyperglycemic conditions in alloxan-induced diabetic mice (Table 2, Figure 2) with disturbances of carbohydrate, fat and protein metabolism as a result of the destruction of pancreatic β cells and insulin deficiency. The impaired metabolism caused by alloxan, as a hyperglycemic agent, led to a gradual reduction in the body weight of mice up to 13.63% during the experimental period (Table 1), which was attributed to the process of dehydration, loss of muscle mass and catabolism of fats and proteins. Thus, diabetic animals showed the following signs of the condition: polydipsia (abnormal thirst), polyuria (increased urine volume), weight loss (due to lean mass loss), asthenia (weakness due to the inability to use glucose as a source of energy), dehydration (due to the animal body’s attempt to get rid of the excess blood glucose because the normal process of storing glucose in the body cells is impaired). During the experiments, four animals from the diabetic control group died, while diabetic animals treated with CA all survived (data not shown). 

The treatment of diabetic animals with CA reversed the metabolism to normal values, confirming that it has an effect in controlling hyperglycemia (Table 2, Figure 1a,b and Figure 2). The weight of healthy animals increased throughout the experimental period and increased by about 13% on day 45 upon completing the experiment (45 days), compared to the initial weight (Table 1). 

Hyperglycemia activates the oxidative pathway through the increased generation of ROS, which in large quantities can lead to cellular damage in lipids, membranes, proteins and DNA [11,12,13,14,15]. Comet assay revealed that alloxan treatment-induced DNA damage in the blood cells of treated animals and that CA can be an effective protector against alloxan-induced DNA damage; CA significantly decreased the % of tail DNA compared to alloxan treatment (Figure 5). On the contrary, in liver and kidney cells we observed a different outline of action. Alloxan in those cells failed to significantly increase DNA damage, but caused significant changes in the level of apoptosis and necrosis compared to the control mice (Figure 8), confirming that high levels of ROS induced severe damage to DNA, proteins and lipids, which could lead to cell death via either apoptotic or necrotic mechanisms (Figure 8). It seems that, through the generation of ROS, alloxan caused damage, not only to pancreatic β-cells, but also to the kidney and liver cells, which is consistent with MDA levels results (Figure 4). In contrast to alloxan, in CA-treated diabetic mice, the percentage of apoptosis and necrosis in the kidney and liver was reduced, confirming its potent antioxidant effect in vivo (Figure 8). Alloxan’s damaging effect on the liver, kidney and pancreatic β-cells was glucose dose-dependent, as indicated by an increase in glucose levels over time (Figure 2), and its effect depended on the balance between the antioxidant capacity of the cell and the toxic effect of alloxan (Figure 4 and Figure 8). Furthermore, our results confirm that the use of CA as an antioxidant, that can easily be supplemented in the diet, may reduce the toxic effect of alloxan-mediated damages of the DNA in the liver, kidney and blood cells (Figure 5) by scavenging reactive radicals, by preventing excessive cell death through apoptosis or necrosis in the liver and kidney cells (Figure 8) and by accelerating regenerative processes and functional characteristics of the kidney and liver, as indicated by serum biochemical parameters (Table 4). In addition to the antioxidant capacity of CA, it significantly reduced glucose levels in healthy and alloxan-induced diabetic groups of mice. These data confirm that CA, as a potent antioxidant and anti-inflammatory compound, can control ROS levels, reducing lipid peroxidation and cellular damage, in part through the regulation of sugar levels, whose high production stimulates free radical formation. Thus, chronic exposure to high concentrations of glucose and fatty acids may cause damage to cells through different mechanisms, but oxidative stress may be a common link in cell dysfunction. The relation between cell survival and cell death is crucial for the normal development and homeostasis of organisms. It has been shown that some tissues such as the heart, kidney and retina are more susceptible to damage in diabetes patients, whereas the liver is more resistant [28], which matches our data (Figure 5 and Figure 8). ROS can both activate and repress NF-κB signaling in a phase- and context-dependent manner. Thus, the NF-κB pathway can have both an anti- and pro-oxidant role in the setting of oxidative stress [29]. According to Eckardstein and Widmann [30], a low amount of NF-κB could be protective and contribute to cell survival by inducing the expression of antiapoptotic genes. More specifically, low/moderate concentrations of ROS can have a beneficial effect by triggering a cellular response to oxidative stress by acting as a cellular messenger in the intracellular signaling cascade wherein it plays a role in the redox regulation of normal physiological functions, as well as in the pathophysiological implications of altered redox regulation in diseases. It seems that differences in hepatocyte and kidney cell responses could be related to the activation of different signaling pathways that may lead to cell death or cell survival [11,15,16]. 

A previous study in our laboratory showed that T1D changed the oxidative balance in the liver and kidney of alloxan-induced diabetic mice, which was characterized by a significant degenerative change in the liver and kidney histology [17,18]. Histologically, the liver section of alloxan-induced diabetic mice showed marked structural alterations in the liver due to the absence of insulin (Figure 6a). Major alterations, such as cellular vacuolization and the apoptosis and necrosis of hepatocytes were partially reversed by CA (Figure 6b). The kidney histopathology data of alloxan-induced diabetic mice showed marked tubular damage and hemorrhages in Bowman’s space due to glomerular damage (Figure 7a). According to our results, kidney damage may be caused by a primary effect associated with hyperglycemia and a secondary effect associated with the inflammatory processes of the diabetic state in mouse kidneys. At the same time, histological studies revealed that alloxan-induced damage to the glomerular and tubular structure was also ameliorated with CA (Figure 7b).

The toxic effect of alloxan on mouse liver and kidney tissues led to a significant increase (*P* < 0.05) in serum transaminases (ALT, AST), alkaline phosphatase (ALP) and LDH activities when compared to the control group (Table 4). High levels of ALT, AST and ALP in the serum of diabetic mice are signs of liver dysfunction and may be related to oxidative stress, insulin resistance and advanced glycosylation end-products [31,32]. Liver dysfunction and the increased activity of liver enzymes are usually regarded as expressions of cellular necrosis, especially in hepatocytes, and a clear indication of cellular leakage and the loss of functional integrity in the cell membrane [2,12,17,18,33]. 

The appearance of LDH indicated cell damage and death after stress, starvation, dehydration, injury, hypoxia, toxin, drugs or chemical poisonings, such as alloxan, which led to cell damage and the loss of the functional integrity of the cell membrane in the liver and other tissues. Therefore, LDH is an indicator of cellular metabolic changes and may be an important target in the pathophysiology and therapy of diabetes. According to Ainscow et al. [33], a significant increase in serum LDH activity in diabetic mice may be due to cellular damage and a persistent inflammatory process. Apart from being a marker for cellular stress and hypoxia, LDH may induce insulin resistance and inhibits insulin action. According to our data (Table 4), the significant increase in LDH activity in the alloxan-treated group was probably related to glucose metabolism, alloxan toxicity and inflammation, as LDH is one of the key terminative enzymes in the sequence of reactions involved in anaerobic glycolysis, promotes glucose breakdown to lactate and is crucial for the production of ATP molecules. In particular, significant weight loss in alloxan-treated mice confirmed that alloxan induced diabetes through the selective destruction of insulin-secreting pancreatic β-cells due to its accumulation through glucose transporter 2 (GLUT2) and thus, effectively minimized glucose uptake into peripheral tissues [34]. 

The treatment of diabetic mice with CA showed improvements in biochemical parameters compared with untreated diabetic mice but a slight increase compared to control animals (Table 4). We observed that CA significantly lowered the alloxan-elevated serum liver enzymes (ALT, AST, ALP, LDH) and blood urea. The above data confirmed the antioxidant, anti-inflammatory, antigenotoxic, protective and regenerative ability of CA on the liver, kidney and other tissues, which in turn confirmed the possibility of its use in the treatment of diabetes and its complications. In addition, according to Jung [25], Jackson et al. [35] and Huang et al. [36], CA, via increasing the amount of hepatic glucokinase, increases the consumption of blood glucose for developing energy or storing the glycogen in the liver. CA and cinnamic acid increased GLUT-2 protein expression by 41.1 and 24.9% of the basal value, respectively [36]. Furthermore, CA successfully regulates and decreases glycosylated hemoglobin levels, exerting long-term diabetic control [37], which is consistent with successful animal survival and the glucose levels at the end of the experiment (Figure 2). 

Other biochemical parameters such as total cholesterol, triglycerides (TG), low-density lipoprotein cholesterol (LDL-C) and very-low-density lipoprotein cholesterol (VLDL-c), as a result of diabetes, significantly increased while high-density lipoprotein cholesterol (HDL-c) decreased (Table 5). It is known that high levels of triglycerides, LDL-c and VLDL-c have been associated with atherogenic indices (AIP, AC, CRR), heart disease and insulin resistance (Figure 6). Such serum lipid biochemical parameters implied that diabetes caused a significant impairment in microvascular and macrovascular systems, liver and kidney functions and dyslipidemia. Apart from atherogenic indicators, we used the TG/HDL-c ratio as the surrogate indicator for insulin resistance, as suggested in recent literature [38]. According to Behiry et al. [38], the TG/HDL-c ratio may be a marker of cardiometabolic risk and cardiovascular disease as well as a useful parameter in hypertensive subjects. In addition to increasing the risk of CVD in people with T1D and high blood glucose levels, over the long run, insulin resistance can also increase the risk of other diabetes-related complications. Some authors [39] suggested that mechanisms, such as oxidative stress, that are associated with hyperglycemia, lipotoxicity and glucotoxicity, as well as inflammation, are also responsible for the development of IR in T1DM.

However, CA leads to a significant reduction in serum TC and LDL cholesterol and significantly elevated serum protein HDL, and it reverses body weight loss (Table 1 and Table 5). CA reduced risk for atherosclerosis (reduced AIP, AC and CRR and increased CPI) through an elevated HDL-c level and had beneficial effects on many cell types, including the endothelium and macrophage foam cells (Table 5). Many scientific data have shown that antioxidants, including CA, have a protective effect against oxidative stress in diabetic animals and patients [16,35,36,37], decrease the FBG level and improve insulin resistance and lipid metabolism (Table 5). According to our data, it seems that CA has the potential to reduce coronary artery disease through the normalization of lipids and preventing lipid oxidation (Table 5). In addition to CA’s effect, HDL may also exert antidiabetogenic functions by inhibiting stress-induced cell death in β-cells and enhancing glucose-stimulated insulin secretion as well as insulin-dependent and insulin-independent glucose uptake into skeletal muscles, adipose tissue and liver [40]. High HDL can also reverse cholesterol transport by scavenging excess cholesterol from peripheral tissues to the liver for its metabolism and excretion [41] and inhibiting LDL oxidation. In addition, HDL can reduce or neutralize the atherogenic effects of oxidized LDL in artery walls. This effect may be increased by CA and its antioxidative capacity, which was confirmed by different in vitro antioxidant assays, including total antioxidant activity through activity in the β-carotene-linoleic assay, reducing power, DPPH• scavenging and metal chelating activity (our unpublished data, [42]). Like other natural phenols, CA is known as a “multifunctional antioxidant” and achieves its protective role through several mechanisms, such as activating and protecting intercellular antioxidant enzymes, as well as through hydrogen atom transfer, single electron transfer and metal chelation [22]. In addition, CA enhances cytoprotective enzymes through Nrf-2 signaling while lowering inflammation through the NF-kB pathway. According to some authors, the effectiveness of CA could be related to the generation of *o*-quinone and the regeneration of CA through the disproportionation of the semiquinone radical initially formed by a reaction involving an antioxidant and a free radical [43]. 

The antioxidant effectiveness of CA is also important in protecting erythrocytes. Increased ROS levels in diabetic erythrocytes affect their function, leading to diminished lifespan, reduced deformability, increased microviscosity, aggregation and adhesiveness of the endothelial layer, enhancing thrombotic events [44]. It is known that hyperglycemia alters the membrane properties of RBC, leading to increased osmotic fragility through decreased Na^+^/K^+^ ATPase levels, which may cause disturbances in intracellular ion balance and thereby, the acceleration of cellular ageing. Increased osmotic fragility can contribute to the disturbances in microvascular circulation observed in diabetes mellitus. Thus, diabetes with chronic hyperglycemia can cause clinical complications, including erythrocyte osmotic fragility and anemia (Table 3, Figure 3a), blindness, hypoxia and organ damage (particularly of the brain, heart, kidneys, nerves, blood vessels and heart), stroke and death [44,45]. 

Mild anemia in diabetic mice is confirmed by a decreased RBC count, hematocrit (Hct) and hemoglobin (Hb) without changes in other parameters except for an increase in MPV, which corresponds to the increase with FBG, as suggested by Shimodaira et al. [46]. It is possible that the main contributors to anemia may be proinflammatory cytokines such as interleukin 6 (IL-6) and tumor necrosis factor α (TNF-α). TNF-α contributes to anemia by inhibiting erythropoietin secretion and/or through direct toxicity on erythroid precursor cells [47], while interleukin-6 induces the production of hepcidin, which binds to ferroportin, thereby blocking the intestinal absorption of iron [48]. 

Increased MPV and platelet size are considered markers and determinants of platelet function and produce higher amounts of the prothrombotic factor thromboxane A2, leading to thrombosis. Platelet aggregation and thrombosis play a key role in the progression of atherosclerosis and consequent cardiovascular complications. Diabetes is characterized by enhanced platelet activation and coagulation proteins and reduced fibrinolytic activity [49]. It seems that CA supplementation in diabetic mice significantly reversed these hematological parameters, as well as platelet activation and thrombosis, reducing microvascular complications (nephropathy, neuropathy and retinopathy) due to platelet dysfunction [50]. This protective effect is compounded by the antioxidant and anti-inflammatory activity of CA, in particular the inhibition of NF-kB and the decreased production of TNF- α, IL-1-β and IL-6 [51]. Thus, we suggested that CA reversed hematological parameters to normal values by attenuating proinflammatory cytokine production and through the secretion of erythropoietin, which stimulates stem cells in the bone marrow to produce red blood cells. CA may participate directly in the amelioration of RBC indices and the correction of the anemic status in diabetes patients and may improve organ and tissue function; reduce fatigue; improve vascular perfusion; maintain glucose homeostasis; assist with wound and tissue repair; and promote cellular proliferation, differentiation and survival [52,53].

Diabetes-induced oxidative stress, due to high glucose concentrations and insufficient antioxidant defense mechanisms, causes damage to erythrocyte membrane proteins via the oxidation or glycation of membrane and cytoskeletal proteins even with a relatively short exposure time. Erythrocytes may be the best models to verify the effects of oxidative conditions in living cells because RBC membranes are more susceptible to alloxan–induced ROS and oxidative damage. The continuous production of ROS, due to high oxygen tension in arterial blood, releases hemoglobin from erythrocytes and creates an oxidized form of hemoglobin, leading to its hem degradation and the release of Fe, which can directly damage the RBC’s membrane through the lipid peroxidation of polyunsaturated membrane fatty acids. H_2_O_2_ can initiate the formation of free radicals in the presence of iron, described by the Haber–Weiss reaction, and converts the polyunsaturated fatty acids to radicals, which propagates a chain reaction of lipid peroxidation in the presence of oxygen. Supplementation with antioxidants such as CA can decrease the occurrence of complications in diabetic animals. In this study, we demonstrated a significant decrease in erythrocyte osmotic fragility and hemolysis (Figure 3a,b) in diabetic mice treated with CA, possibly through its hypoglycemic, antioxidative and scavenging activity (Figure 4). The anti-inflammatory and antioxidant properties of CA were also demonstrated by reduced leukocyte counts in diabetes-treated mice. A moderate increase in the leukocyte count in diabetic model mice also indicates a low degree of inflammation, most commonly associated with a slight increase in neutrophil percentage and the prolonged circulation times of neutrophils and monocytes (Table 3), as suggested by Mahmoud [31].

Summarizing our data with data from other authors [16,20,21,22,35,36,37,38,52,53], we can conclude that CA acts against diabetes and hyperglycemia through various mechanisms, including CA exerting antidegenerative effects and promoting the survival of islets in animal models; decreasing the serum level of FBS in diabetic animals; regulating β-cell and adipocyte GLUT4 performance; effectively inhibiting the activity of α-amylase and α-glucosidase in the gastrointestinal tract and increasing the activity of glucokinase in hepatocytes; inhibiting the activity of glucose-6 phosphatase and phosphoenolpyruvate carboxykinase; reducing glycosylated hemoglobin levels; exerting long-term diabetic control; improving the consumption of glucose and glycogen syntheses; effectively preventing cholesterol biosynthesis and suppressing the activity of lipogenesis; inhibiting iron-induced hypercholesterolemia; and increasing plasma insulin, C-peptide and leptin levels.

## 4. Materials and Methods

### 4.1. Chemicals and Apparatus

Alloxan was purchased from Sigma-Aldrich Chemical Co. (Saint Louis, MS, USA). Caffeic acid (CA, 3,4-Dihydroxy-cinnamic acid, C_15_H_10_O_4_; Mt:180,2 purity ≥ 98%) was purchased from Aldrich-Chemie, Milwaukee, WI, USA, while the glucometer (Accu-Chek Advantage II) and compatible blood glucose test strip were purchased from Roche, Mannheim, Germany. For absorbance measurements, a Stat Fax 3200 (Awareness Technologies, Westport, CT, USA) microplate reader and a Perkin Elmer Lambda 25 spectrophotometer (Perkin Elmer, Waltham, MA, USA) were used. 

### 4.2. Animals

Male Swiss albino mice two to three months old, weighing 20 to 25 g, obtained from the Department of Animal Physiology, Faculty of Science, University of Zagreb, were used in this study. The animals were kept in individual cages during the experiment and under standard conditions (temperature 25 ± 3 °C, relative humidity 55 ± 10% and 12 h light and 12 h dark). They were fed a standard laboratory diet (4 RF 21, Mucedola, Settimo Milanese, Italy) and tap water ad libitum. Maintenance, animal housing conditions, handling and care of all experimental animals were carried out according to the guidelines in force in the Republic of Croatia (Animal Welfare Act, OG 19/1999) and carried out in compliance with the Guide for the Care and Use of Laboratory Animals, DHHS Publ. # (NIH) 86-123. All the guidelines enforced in Croatia for mice are in accordance with the internationally accepted principles for laboratory animal use and care as found in the European Community guidelines (EEC Directive of 1986; 86/609/EEC). The Ethics Committee of the Faculty of Science (University of Zagreb, Croatia) approved the study (approval code: 3804-508-09-175).

### 4.3. Induction of Experimental Diabetes, Experimental Design and Determination of Serum Glucose Level

Diabetes was induced in male Swiss albino mice through a single intravenous injection of alloxan monohydrate (75 mg kg^−1^, *iv*) in a total volume of 0.5 mL of saline solution prepared freshly. Fasting blood glucose level (FBG) was tested before alloxan injection and 48 h after treatment to monitor diabetogenesis. After 48 h, the animals with a blood glucose level above 11 mmol L^−1^ were selected for the study (diabetic mice) and then treated with CA. FBG level was determined by a glucometer (Accuchek Advantage II) and a compatible test strip.

Sixty mice were randomly divided into four groups with 15 mice in each group, as follows: 

Group (1): control animals (normal healthy animals) who received 0.5 mL distilled water intraperitoneally (*ip*) daily for seven days;

Group (2): normal healthy animals treated with caffeic acid (CA); CA was dissolved in water and then injected intraperitoneally into mice daily at a dose of 50 mg kg^−1^ for seven days [54,55]. The applied dose corresponds to a human intake of CA in the diet [56,57];

Group (3): diabetic control group, injected *iv* with alloxan at a single dose of 75 mg kg^−1^ body weight and served as untreated diabetic group;

Group (4): diabetic group, treated with CA (50 mg kg^−1^ *ip* daily) for seven days, starting two days after the alloxan injection, and served as the CA-treated diabetic group. 

Six mice from each group were used on the 10th day after alloxan injection. On the 10th day after anesthesia (isoflurane, 2% in a flow of oxygen), the following variables were analyzed: body weight, blood glucose level, hematological and serum biochemical assay for the evaluation of renal and hepatic function, lipid profile parameters and atherogenic indices, erythrocyte hemolysis test, erythrocyte osmotic fragility, lipid peroxidation, the alkaline comet assay, histopathological analysis and apoptosis/necrosis cell death. 

Blood samples were collected into a tube containing EDTA for the estimation of blood glucose levels, erythrocyte fragility, hemolysis test and hematological parameters, while other blood parts were kept for clotting at laboratory temperature for 30 min and centrifuged at 2200 rpm for 10 min for serum separation. The obtained serum was used to determine the levels of urea, glucose, total protein, lipid profile parameters and the activities of the enzymes alanine aminotransferase (ALT), aspartate aminotransferase (AST) and lactate dehydrogenase (LDH). The liver, kidney and brain were dissected, washed with ice-cold saline, dried, weighed and subjected to lipid peroxidation (LPO) assessment by measuring malondialdehyde (MDA) formation through the thiobarbituric acid method described below. 

The remaining animals, i.e., nine animals from each group, were used for body weight survival analysis and fasting blood glucose (FBG) level monitoring. FBG levels were monitored periodically from the tail vein of each fasted mouse with the tail prick method using a glucometer (Accu-Chek Advantage II, Roche, Germany). Blood glucose levels were expressed in mmol L^−1^. 

### 4.4. Effect of Caffeic Acid on Body Weight in Alloxan-Induced Diabetic Mice 

During the 45-day study period, the mice were weighed every seven days using an electronic balance, and their body weights were recorded. From this data, the percentage of body weight change was calculated according to Equation (1):

(1) $$\;\mathrm{Weight}\;\mathrm{change}\;\left(\%\right)=\frac{\left(\mathrm{final}\;\mathrm{weight}-\mathrm{initial}\;\mathrm{weight}\right)\times 100}{\mathrm{final}\;\mathrm{weight}}$$

### 4.5. Assessing the Hypoglycemic and Antihyperglycemic Effect of Single and Repeated Doses of Caffeic Acid on Fasting Blood Glucose Levels (FBG)

FBG levels were then measured for each tested mouse after a single or repeated dose of CA at different time intervals. After overnight fasting, FBG levels were measured for each tested mouse, after a single dose just before treatment (at 0 h) and then at 1, 2, 4, 6 and 8 h post-treatment. The effects of the repeated dose of CA on diabetic mice were measured at different time intervals (2, 3, 5 and 10 days). The whole blood of each experimental mouse was drawn from the tail vein following overnight fasting at the same time and in the first 10 days 1 h after treatment. The percentage of glycemic variation within a group was calculated as a time (t) function by applying Equation (2):

(2) $$\%\;glycemic\;change=\frac{\left(G_{0}-G_{1}\right)\;}{G_{0}}\times 100$$

where G_0_ and G_1_ represent glycemic values before and at one, two, four, six and eight hours after caffeic acid treatment, respectively.

The percentage of blood glucose level reduction in relation to the corresponding controls was calculated according to Equation (3):

(3) $$\mathrm{Percent}\;\mathrm{lowering}\;\mathrm{of}\;\mathrm{blood}\;\mathrm{glucose}\;\mathrm{level}\;=\left(\frac{\left(1-Ge\right)}{Gc}\right)\times 100$$

where *Ge* and *Gc* represent the blood glucose concentration in CA-treated healthy or CA-treated diabetic mice and corresponding control mice (healthy control or alloxan-induced diabetic mice), respectively.

### 4.6. Hematological and Serum Biochemical Assay for the Evaluation of Renal and Hepatic Function

For hematological parameters, blood samples were collected from axillary blood vessels into EDTA tubes under light anesthesia. White blood cells (WBC), red blood cells (RBC), hematocrit (HCT), hemoglobin (HB), mean cell volume (MCV), mean cell hemoglobin (MCH) and mean cell hemoglobin concentration (MCHC) were measured on a Hematology Analyzer Cell-Dyn 3700 (Abbott, Abbott Park, IL, USA).

Other blood sample parts were collected and immediately placed on ice prior to the isolation of serum through centrifugation at 2200 rpm for 10 min. Serum was used for the estimation of the serum liver enzyme activity and kidney function. We analyzed total protein, glucose, urea, alkaline phosphatase (ALP), aspartate and alanine aminotransferases (AST and ALT) and lactic dehydrogenase (LDH). Biochemical parameters were measured by an automatic cell counter Alcylon 300 (Abbott, Chicago, IL, USA).

### 4.7. Estimation of Lipid Profile Parameters in Serum

On the tenth day, 24 h after the last treatment and overnight fasting, blood was taken from the axillary blood vessels after anesthetizing the animals, and the serum was extracted after coagulation. The unhemolyzed serum was collected and frozen at −80 °C until further processing. Serum lipid parameters such as total cholesterol (TC), triglyceride (TG), a low-density lipoprotein (LDL) and high-density lipoprotein (HDL) were evaluated according to the recommendations of the IFCC methods in enzymology and were done with commercial kits (Sigma-Aldrich, Germany) on a Hitachi 717 automatic analyzer (Hitachi, Japan). The LDL-c and very-low-density lipoprotein-cholesterol (VLDL-c) concentrations were calculated from the Friedewald equation: LDL-cholesterol: [LDL-c] = [TC] − [HDL-c] − [TG/5] and VLDL-c = TG/5, according to the manufacturer’s instructions [58]. A detailed description of the methods is described in the paper [24].

### 4.8. Atherogenic Risk Index

The atherogenic risk index (ARI) was calculated as previously reported [24], using Equation (4):

(4) $$\mathrm{ARI}\;\mathrm{or}\;\mathrm{Atherogenic}\;\mathrm{coefficient}\;(\mathrm{AC}):\;ARI=\frac{\left(TC-HDL-c\right)}{HDL-c}$$

### 4.9. Percentage Protection

The percentage protection of the CA-treated DM group against atherogenicity was calculated with Equation (5):

(5) $$Percentage\;protection=\frac{\left(ARI_{Negative\;control\;group\;}-ARI_{Treated\;group\;}\right)}{ARI_{Negative\;control\;group}}\times 100$$

where:

Negative control group = untreated DM group.

Treated group = caffeic acid-treated DM group.

### 4.10. Atherogenic Risk Predictor Indices (ARPI)

After determining the concentration in mg dL^−1^ of the TC, TG, HDL-c and LDL-c fractions, atherogenic risk predictor indices ARPI-1, ARPI-2, ARPI-3 (ARPI-1 or atherogenic index of plasma, AIP); ARPI-2, the relation between LDL-c and HDL-c; ARPI-3 or the cardiac risk ratio (CRR); cardioprotective index (CPI); and the indicator of insulin resistance, as the TG/HDL-c ratio was calculated using the values of lipid profile parameters as described in [24] by Equations (6)–(10):

(6) $$\mathrm{ARPI}-1\;\mathrm{or}\;\mathrm{the}\;\mathrm{atherogenic}\;\mathrm{index}\;\mathrm{of}\;\mathrm{plasma}\;(\mathrm{AIP}):\;\mathrm{ARPI}-1=\log\frac{TG}{HDL-c}$$

ARPI-2 = LDL-c/HDL-c(7)

(8) $$\mathrm{ARPI}-3\;\mathrm{or}\;\mathrm{the}\;\mathrm{cardiac}\;\mathrm{risk}\;\mathrm{ratio}\;(\mathrm{CRR}):\;ARPI=\frac{TC}{HDL-c}$$

(9) $$\mathrm{Cardioprotective}\;\mathrm{index}\;(\mathrm{CPI}):\;CPI=\frac{HDL-c}{LDL-c}$$

(10) $$\mathrm{An}\;\mathrm{indicator}\;\mathrm{of}\;\mathrm{insulin}\;\mathrm{resistance}\;(\mathrm{IR}):\;IR=\frac{TG}{HDL-c}$$

### 4.11. Erythrocyte Hemolysis Test

The RBC membrane is altered by free radical-induced oxidative stress, and the reduced antioxidant activity in erythrocytes may increase their sensitivity to hydrogen peroxide-induced hemolysis. Hydrogen peroxide-induced hemolysis was measured by the method from Greenberg et al. [59], with some modifications. A 2 mL erythrocyte sample was incubated with 100 μM of CA for 1 h at 37 °C under aerobic conditions. The cells were then centrifuged, washed and resuspended in PBS. Hydrogen peroxide (H_2_O_2_) was added to a final concentration of 100 μM, and the erythrocytes thus treated were incubated at 37 °C. Samples of 0.2 mL were taken at 30-min intervals starting from 0 min (immediately after adding H_2_O_2_) to 240 min. Then, they were diluted in 2 mL of PBS and centrifuged. We measured the hemolysis scales spectrophotometrically at 540 nm wavelength. The samples thus obtained were compared with controls prepared in an identical manner, except that the H_2_O_2_ solution was replaced with distilled water, and the extracellular hemoglobin contained in the samples was completely hemolyzed. The erythrocyte samples without preincubation with CA were also tested. We expressed the percentage of hemolysis according to Equation (11):

(11) $$\mathrm{Hemolysis}\;(\%)=\left(\frac{A}{B\;}\right)\times 100$$

where *A* is the absorbance of the sample at 540 nm, and *B* is the absorbance of the fully hemolyzed reference sample at 540 nm. 

### 4.12. Measurement of Erythrocyte Osmotic Fragility

RBC deformability is related to the osmotic fragility of RBCs and is based on the RBC resistance to lysis as a function of a decreasing NaCl concentration. The osmotic fragility of erythrocytes was determined using the modification of a method described by Ambali et al. [60]. A set of glass tubes was prepared, with 9 mL of 0.9%, 0.8%, 0.7%, 0.6%, 0.5%, 0.4%, 0.3%, 0.2%, 0.1% and 0.0% NaCl solution in each. A whole blood sample was mixed using a glass stirring rod to maintain a homogenous cell suspension. Next, 0.1 mL of each blood sample was pipetted into each tube in a set of prepared NaCl solutions. Tubes were covered, mixed by turning them upside-down a couple of times, and incubated at room temperature for 30 min, then centrifuged for 10 min at 2200 rpm. A supernatant of each tube was transferred to cuvettes using a Pasteur pipette. The optical density of the supernatant was determined spectrophotometrically at 540 nm using a Spectrophotometer Libra S22 (Biochrom, Cambridge, UK). The experiments were performed in triplicate and the results expressed as % hemolysis compared to the positive control group (100% hemolysis) in distilled water.

### 4.13. Lipid Peroxidation

Samples of liver, brain and kidneys were homogenized in a 50 mM phosphate buffer (pH 7.0, 100 mg tissue in 1 mL buffer) and centrifuged at 10,000 rpm for 15 min. For biochemical analyses, the supernatant was used. Lipid peroxidation in tissue was determined according to the method described by Oršolić et al. [15,55] and expressed as nmol of formed malondialdehyde (MDA) mg protein^−1^. The concentration of protein was determined according to Lowry at al. [61] using bovine serum albumin as the standard.

### 4.14. The Alkaline Comet Assay

The comet assay is a sensitive method for detecting primary DNA damage in individual cells in experimental animals [14,15]. In order to analyze DNA damage after alloxan treatment and combined treatment with CA, the alkaline version of the comet assay was used, as described by Singh et al. [62], with minor modifications described in our previous studies [14,15]. After the animals were sacrificed, samples of whole blood cells, liver and kidneys were taken, immersed in chilled homogenization buffer and mechanically homogenized to obtain cell suspensions. Afterwards, the whole blood or cell suspension of other organs was embedded in agarose (Sigma) matrix and the cells were lysed (2.5 M NaCl, 100 mM Na_2_EDTA, 10 mM Tris, 1% sodium sarcosinate (Sigma), 1% Triton X-100 (Sigma), 10% DMSO (Kemika), pH 10) at 4 °C for 1 h. Next, the slides were placed into the alkaline solution (300 mM NaOH, 1 mM Na_2_EDTA, pH 13) at 4 °C for 20 min and subsequently electrophoresed for another 20 min at 1 V/cm. Finally, the slides were neutralized in 0.4 M Tris buffer (pH 7.5) for 3 × 5 min, stained with ethidium bromide (Sigma; 20 µg/mL), and analyzed at 250× magnification under an epifluorescence microscope (Zeiss, Oberkochen, Germany) connected to software (Comet Assay II; Perceptive Instruments Ltd., Haverhill, Suffolk, UK). The % of tail DNA, as the comet assay descriptor, was used to quantify the level of DNA damage, and a total of 100 comets were examined from each slide.

### 4.15. Histopathological Analysis

Liver and kidney tissues from diabetic control mice treated and diabetic mice treated with CA were fixed in 10% neutral buffered formalin for 24 h. After fixation, tissues were dehydrated in a graded alcohol series and, after chloroform treatment, embedded in Paraplast. Deparaplasted 5–6 μm thick sections were stained with hematoxylin and eosin (HE), following standard protocol, and examined under a light microscope (Nikon Eclipse E600, Nikon, Tokyo, Japan). Stained slides were examined under a light microscope (Nikon Eclipse E600) at 100, 200, 400 and 1000× magnification. Liver tissue sections were examined for lymphocyte infiltrations, vacuolization, necrosis and apoptosis. To determine the percentage of apoptotic cells, two hundred cells in randomly selected microscopic fields of vision was examined. Kidney sections were examined for lymphocyte infiltrations, changes in renal corpuscles, renal tubules and apoptosis and necrosis. Photomicrographs were taken by a digital camera (Nikon DMX1200, Nikon, Tokyo, Japan), and imaging software Lucia G 4.80 (Laboratory Imaging Ltd., Prague, Czech Republic).

### 4.16. Statistical Analysis

Comet assay results were evaluated using the Statistica 13.0 package (StaSoft, Tulsa, OK, USA). Each sample was characterized for the extent of DNA damage by considering the mean ± SE (standard error of the mean). In order to normalize the distribution and equalize the variances, a logarithmic transformation of data was applied. Multiple comparisons between groups were done on log-transformed data. A post-hoc analysis of differences was done by a Scheffé test. Other data were analyzed through a nonparametric Kruskal–Wallis test, and further analyses of the differences between the groups were made with multiple comparisons of mean ranks for all groups. Values of *P* lower than 0.05 were considered statistically significant.

## 5. Conclusions

To conclude, diabetes mellitus caused by alloxan induced a significant elevation of BGL; TG; TC; LDL; VLDL cholesterol; liver enzymes; serum blood urea; MDA level; and DNA damage in peripheral blood cells, liver and kidney, and a significant decrease in the serum protein, HDL and body weight. CA treatment in diabetic mice revealed a protective effect on the liver and kidney, as well as the hypoglycemic and hypolipidemic properties of CA, and suggested that, in diabetes patients, CA is a safe and potent agent that acts as an effective antioxidant in reducing serum glucose and LDL, leading to lower atherogenic indices. CA successfully regulates the hematological and biochemical abnormalities associated with diabetes that lead to weight gain, long-term diabetic control and animal survival. The excellent recovery of liver and renal function can be explained by the antioxidative, anti-inflammatory and regenerative capability of CA. CA could be used as a part of healthy diet supplement to protect against diabetes and its complications. 

## Figures and Tables

**Table 1:** Effect of caffeic acid on body weight changes in healthy and alloxan-induced diabetic mice.

Day	Treatments ^a^ (Mean ± SEM)			
	Control (H) (% Change)	Caffeic Acid (CA) (% Change)	Alloxan (D) (% Change)	D + CA (% Change)
0	26.00 ± 1.10	25.00 ± 1.80	25.00 ± 1.65	25.00 ± 1.50
3	27.80 ± 2.00 (6.47)	25.70 ± 1.60 (3.10)	24.10 ± 1.80 (−3.73)	24.80 ± 1.70 (−0.80)
10	28.80 ± 2.20 (9.72)	26.60 ± 1.90 (6.70)	22.30 ± 1.70 (−12.11) ∗	23.40 ± 1.30 (−6.83) ∗
17	29.40 ± 1.32 (11.56)	27.10 ± 1.70 (8.30)	22.80 ± 1.50 (−9.65) ∗	24.80 ± 1.50 (−0.80) ∗
24	29.40 ± 1.35 (11.56)	27.20 ± 1.50 (8.50)	23.50 ± 1.40 (−6.40) ∗	26.70 ± 1.25 (6.36)
31	29.70 ± 1.50 (12.45)	27.50 ± 1.40 (9.90)	22.20 ± 1.70 (−12.61) ∗∗	26.00 ± 1.90 (3.84) ^◊^
38	29.50 ± 1.90 (11.86)	27.20 ± 1.70 (8.70)	22.10 ± 1.20 (−13.12) ∗∗	26.20 ± 1.10 (4.58) ^◊^
45	29.80 ± 1.70 (12.75)	27.30 ± 1.65 (9.30)	22.00 ± 1.20 (−13.63) ∗∗	26.20 ± 2.30 (4.58) ^◊^

^a^ Diabetic mice were injected *iv* with alloxan at a single dose of 75 mg kg^−1^ body weight. The treatment of diabetic animals with caffeic acid started on the second day after induction of diabetes with at a dose of 50 mg kg^−1^ *ip* for 7 consecutive days. Results are presented as the percentage of body-weight change calculated by the formula described in Material and Methods; *n* = 9 for each treatment. ∗ Statistically significant compared to healthy (H) control group (∗ *P* < 0.05; ∗∗ *P* < 0.01). ^◊^ Statistically significant compared to diabetic (D) control group (^◊^ *P* < 0.05). Abbreviations: H, control group (normal healthy animals); CA, caffeic acid-treated healthy animals; D, diabetic control group (diabetic model animals); D + CA, diabetic group treated with CA.

**Table 2:** Effect of a single dose of caffeic acid on blood glucose level in healthy and alloxan-induced diabetic mice.

Exp. Group ^a^	Blood Glucose Level (mmol L^−1^)					
	0 h	1 h	2 h	4 h	6 h	8 h
**Control (H)**	5.70 ± 0.50	5.65 ± 0.35	5.73 ± 0.09	5.69 ± 0.20	5.71 ± 0.39	5.69 ± 0.41
**Caffeic acid (CA)**	5.81 ± 0.40	5.69 ± 0.50	5.46 ± 0.35	5.39 ± 0.07 *^,^^Δ^	5.53 ± 0.12	5.57 ± 0.37
**Alloxan (D)**	13.41 ± 1.51	13.45 ± 1.50	13.20 ± 0.97	13.53 ± 1.30	13.45 ± 1.51	13.6 ± 1.53
**D + CA**	13.27 ± 1.50	12.93 ± 1.47	12.37 ± 1.53	11.95 ± 0.79 ^◊,^^∇^	12.39 ± 0.65	12.93 ± 0.43

^a^ Diabetic mice were injected *iv* with alloxan at a single dose of 75 mg kg^−1^ body weight. The treatment of healthy control animals or diabetic animals with a single dose of caffeic acid started on the second day after the induction of diabetes with a dose of 50 mg kg^−1^ *ip.* Results are presented as mean ± SEM; *n* = 9 for each treatment. ∗ Statistically significant compared to the healthy (H) control group (∗ *P* < 0.05). ^◊^ Statistically significant compared to the diabetic (D) control group (^◊^ *P* < 0.05). ^Δ^ Statistically significant compared to the initial glucose level (at 0 h) in the CA treatment of healthy animals (^Δ^ *P* < 0.05). ^∇^ Statistically significant compared to the initial glucose level (at 0 h) in the D + CA treatment of diabetic animals (^∇^ *P* < 0.05). Abbreviations: H, control group (normal healthy animals); CA, caffeic acid-treated healthy animals; D, diabetic control group (diabetic model animals); D + CA, diabetic group treated with CA.

**Table 3:** Effect of caffeic acid on hematological parameters in healthy and alloxan-induced diabetic mice.

Parameters	Treatments ^a^ (Mean ± SEM)			
	Control (H)	Caffeic Acid (CA)	Alloxan (D)	D + CA
**WBC (×10^9^ L^−1^)**	4.80 ± 1.01	4.90 ± 1.50	6.94 ± 0.65	4.41 ± 1.31
**Mononuclear cells (%)**	67.06 ± 4.95	65.13 ± 3.07	48.25 ± 8.66	51.73 ± 4.91
**Polymorphonuclear cells (%)**	32.94 ± 5.08	35.15 ± 3.08	51.75 ± 8.66	48.27 ± 5.17
**RBC (×10^12^ L^−1^)**	9.13 ± 0.32	9.30 ± 0.29	7.93 ± 0.44 ∗	9.43 ± 0.27 ^◊^
**HGB (g L^−1^)**	135.55 ± 5.35	140.00 ± 4.37 ^◊^	117.68 ± 7.58 ∗	136.20 ± 3.67 ^◊^
**HCT (L L^−1^)**	0.44 ± 0.02	0.47 ± 0.25 ^◊^	0.39 ± 0.02 ∗	0.4502 ± 0.01 ^◊^
**MCV (fL)**	97.20 ± 1.91	96.80 ± 2.91	95.28 ± 1.27	95.80 ± 1.50
**MCH (pg)**	29.70 ± 0.26	28.90 ± 2.13	28.50 ± 1.20	28.90 ± 0.41
**MCHC (g L^−1^)**	616.70 ± 6.43	610.00 ± 10.54	606.00 ± 20.70	603.00 ± 7.74
**RDW/%CV**	24.00 ± 3.01	24.30 ± 0.70	25.63 ± 1.16	23.60 ± 1.78
**PLT (×10^9^ L^−1^)**	1434.50 ± 7.75	1237.00 ± 9.73	1554.30 ± 14.79	1321.50 ± 20.90
**MPV/fL**	9.76 ± 0.67	9.67 ± 0.30	11.55 ± 0.68	9.92 ± 0.63

^a^ Diabetic mice were injected *iv* with alloxan at a single dose of 75 mg kg^−^^1^ body weight. The treatment of diabetic animals with caffeic acid started on the second day after the induction of diabetes with a dose of 50 mg kg^−^^1^ *ip* for 7 consecutive days. Results are presented as mean ±SEM; *n* = 6 for each treatment. ∗ Statistically significant compared to healthy (H) control group (∗ *P* < 0.05). ^◊^ Statistically significant compared to diabetic (D) control group (^◊^ *P* < 0.05). Abbreviations: H, control group (normal healthy animals); CA, caffeic acid-treated healthy animals; D, diabetic control group (diabetic model animals); D + CA, diabetic group treated with CA; WBC, leukocytes; RBC, erythrocytes; HGB, hemoglobin; HCT, hematocrit; MCV, average volume of red blood cells; MCH, the average amount of hemoglobin in red blood cells; MCHC, mean concentration of hemoglobin in red blood cells; PLT, platelets; MPV, mean platelet volume.

**Table 4:** Effect of caffeic acid on biochemical parameters in healthy and alloxan-induced diabetic mice.

Parameters	Treatments ^a^ (Mean ± SEM)			
	Control (H)	Caffeic Acid (CA)	Alloxan (D)	D + CA
**ALP (U L^−1^)**	53.75 ± 7.50	51.37 ± 3.21	98.75 ± 5.89 ∗	71.25 ± 20.15
**ALT (U L^−1^)**	66.25 ± 5.09	59.26 ± 4.09	81.25 ± 9.46 ∗	57.25 ± 10.35
**AST (U L^−1^)**	186.25 ± 11.72	184.13 ± 12.54	253.75 ± 17.94 ∗	193.75 ± 11.70
**LDH (U L^−1^)**	1433.51 ± 25.71	1298.48 ± 29.46 ^◊^	2487.50 ± 37.55 ∗∗	986.61± 27.55 ^◊^
**UREA (mmol L^−1^)**	7.50 ± 0.92	5.90 ± 1.40	11.66 ± 3.65 ∗∗	7.88 ± 1.43
**TP (g L^−1^)**	48.50 ± 4.38	49.10 ± 3.10	40.50 ± 1.96	52.63 ± 4.03 ^◊^
**GLU (mmol L^−1^)**	5.70 ± 0.64	5.60 ± 0.70 ^◊◊^	19.41 ± 4.55 ∗∗	9.65 ± 2.66 ^◊^

^a^ Diabetic mice were injected *iv* with alloxan at a single dose of 75 mg kg^−^^1^ body weight. The treatment of diabetic animals with caffeic acid started on the second day after the induction of diabetes with a dose of 50 mg kg^−^^1^ *ip* for 7 consecutive days. Results are presented as mean ±SEM; *n* = 6 for each treatment. ∗ Statistically significant compared to healthy (H) control group (∗ *P* < 0.05; ∗∗ *P* < 0.01). ^◊^ Statistically significant compared to diabetic (D) control group (^◊^ *P* < 0.05; ^◊◊^ *P* < 0.01). Abbreviations: H, control group (normal healthy animals); CA, caffeic acid-treated healthy animals; D, diabetic control group (diabetic model animals); D + CA, diabetic group treated with CA; AST, aspartate aminotransferase; ALT, alanine aminotransferase; ALP, alkaline phosphatase; LDH, lactate dehydrogenase; TP, total proteins, GLU, glucose.

**Table 5:** Effect of caffeic acid on serum lipids biochemical parameters, atherogenicity and atherogenic risk predictor indices in healthy and alloxan-induced diabetic mice.

Parameters	Control (H)	Caffeic acid (CA)	Alloxan (D)	D + CA
TC (mg dL^−1^)	95.35 ± 5.96	93.7 ± 7.37 ^◊◊^	166.50 ± 7.54 ∗∗	130.28 ± 2.31 ∗^,^^◊,Δ^
TG (mg dL^−1^)	92.96 ± 3.54	89.17 ± 4.17 ^◊◊^	152.00 ± 5.89 ∗∗	119.09 ± 4.45 ∗^,^^◊,Δ^
HDL-c (mg dL^−1^)	38.90 ± 2.05	39.33 ± 3.35 ^◊^	31.12 ± 2.73 ∗	46.42 ± 0.97 ^◊^
LDL-c (mg dL^−1^)	37.85 ± 1.17	36.53 ± 2.7 ^◊◊^	114.75 ± 3.35 ∗∗	71.66 ± 1.35 ∗∗^,^^◊,ΔΔ^
VLDL-c (mg dL^−1^)	18.59 ± 0.55	17.83 ± 1.70 ^◊◊^	30.40 ± 1.57 ∗∗	23.81 ± 0.35 ∗^,^^◊,Δ^
TC (mg dL^−1^)	95.35 ± 5.96	93.7 ± 7.37 ^◊◊^	166.50 ± 7.54 ∗∗	130.28 ± 2.31 ∗^,^^◊,Δ^
ARI (AC) = [(TC-HDL-c)/HDLc]	1.45 ± 0.30	1.38 ± 0.22 ^◊^	4.3540 ± 0.72 ∗	1.80 ± 0.60 ^◊^
ARPI-1 (AIP) = [log (TG/HDL-c)]	0.38 ± 0.04	0.35 ± 0.05 ^◊^	0.69 ± 0.08 ∗	0.41 ± 0.06 ^◊^
ARPI-2= [LDL-c/HDL-c]	0.86 ± 0.05	0.80 ± 0.07 ^◊^	4.89 ± 0.57 ∗	2.20 ± 0.19 ∗^,^^◊,^^Δ^
ARPI-3 (CRR) = [TC/HDL-c]	2.45 ± 0.13	2.38 ± 0.23 ^◊^	5.35 ± 0.93 ∗	2.80 ±0.610 ^◊^
CPI = HDL-c/LDL-c	1.03 ± 0.180	1.07 ±0.163	0.27 ± 0.02 ∗	0.65 ± 0.05 ^◊^
IR = [TG/HDL-c]	2.39 ± 0.51	2.27 ± 0.130	4.89 ± 0.74 ∗	3.42 ± 0.33

^a^ Diabetic mice were injected *iv* with alloxan at a single dose of 75 mg kg^−1^ body weight. The treatment of diabetic animals with caffeic acid started on the second day after the induction of diabetes at a dose of 50 mg kg^−1^ *ip* for 7 consecutive days. Results are presented as mean ± SEM; *n* = 6 for each treatment. ∗ Statistically significant compared to healthy (H) control group (∗ *P* < 0.05; ∗∗ *P* < 0.01). ^◊^ Statistically significant compared to diabetic (D) control group (^◊^ *P* < 0.05; ^◊◊^ *P* < 0.01) ^Δ^ Statistically significant compared to CA group (^Δ^ *P* < 0.05; ^ΔΔ^ *P* < 0.01). Abbreviation: H, control group (normal healthy animals); CA, caffeic acid-treated healthy animals; D, diabetic control group (diabetic model animals); D + CA, diabetic group treated with CA; TC, total cholesterol; TG, total triglyceride; HDL-c, high-density lipoprotein cholesterol; LDL-c, low-density lipoprotein cholesterol; VLDL-c, very-low-density lipoprotein cholesterol. ARI, atherogenic risk index (ARI), AC, atherogenic coefficient; ARPI-1, 2, 3, atherogenic risk predictor index 1,2, 3; AIP, atherogenic index of plasma; CRR, cardiac risk ratio; IR, insulin resistance. ARPI-2 = [LDL-c/HDL-c] ratio > 2.3 is atherogenic and undesirable; ARPI-3 (CRR) = [TC/HDL-c] ratio >3.33 is atherogenic and undesirable.

## Data Availability

The orginal contributions generated for this study are included in the article; father inquiries can be directed to the corresponding author.

## Sample Availability

The compounds are not available from the authors.
